# Supplementary material for: Diagnostic Significance of Influenza Symptoms and Signs, and Their Variation by Type/Subtype, in Outpatients Aged ≥ 15 Years: Novi Sad, Serbia
Source: Viruses. 2025 Feb 16;17(2):272. doi: 10.3390/v17020272 (PMC11860240; doi:10.3390/v17020272)
Supplement: Supplementary file 1 [file viruses-17-00272-s001.zip › Suppl. S1.pdf]

Section S1

Performance of variables related to influenza by age categories during 2022/23 influenza season

Patients aged 15-29 years

Fever (≥ 38°C)**,** malaise**,** cough and headache all exhibited relatively high sensitivity (96.43%, 96.43%, 92.86%, and 92.86%, respectively), but their specificity was lower. Nasal congestion had the highest sensitivity (100%), but very low specificity (16.67%). The highest specificity was detected for the shortness of breath (94.05%), and clinical signs of pneumonia – auscultatory (98.81%), but their sensitivities were low (10.71% and 0%, respectively).

Vaccination in this age group was negligible variable.

Asthma showed a low sensitivity (7.14%) and very high specificity (96.43%). It has high accuracy (0.741), meaning it helps detect influenza to a limited extent while being highly specific in excluding it.

Diabetes mellitus type 1 had a high specificity (97.62%) but sensitivity was 0%, indicating it does not aid in identifying influenza. In order other chronic diseases, there were moderate sensitivity (7.14%) and good specificity (89.29%), contributing to moderate accuracy (0.688), suggesting a minor role in diagnosing influenza.

The information that a patient lives in a household with children aged 7 to 14 years demonstrated a sensitivity of 25.00% and a specificity of 80.95%, resulting in moderate accuracy (0.670). In contrast, living in a household with children aged 15 to 18 years was associated with lower sensitivity (3.57%) but very high specificity (91.67%) and also moderate accuracy (0.696). Contact with someone who had flu-like symptoms seven days before testing had high sensitivity (85.71%) with moderate specificity (34.52%), and accuracy of 0.473. Smoking had a moderate sensitivity (17.86%), moderate specificity (78.57%), and the accuracy of 0.634. Alcohol consumption showed moderate sensitivity (21.43%) and very high specificity (90.48%), contributing to a relatively high accuracy (0.732).

Use of buses for transportation had very high sensitivity (85.71%) but low specificity (15.48%), leading to a low accuracy (0.330). Use of TAXI for transportation with moderate sensitivity (60.71%) and moderate specificity (38.10%), this factor showed low accuracy (0.438).

Considering the period 1.12.22–14.2.2023 in order to influenza detection, there was a high sensitivity (71.43%) and moderate specificity (57.14%), resulting in moderate accuracy (0.607). In other hand, the period 15.2.2023–30.4.2023 showed lower sensitivity (28.57%) and specificity (42.86%).

Patients aged 30-64

In patients aged 30–64 years, a good balance between sensitivity and specificity was observed for fever (≥ 38°C), myalgia, and chills. The sensitivity, specificity, and accuracy were 85.42%, 75.59%, and 0.772 for fever; 66.67%, 74.80%, and 0.735 for myalgia; and 58.33%, 76.77%, and 0.738 for chills, respectively.

The highest specificity values were noted for gastrointestinal symptoms and signs (loss of appetite, abdominal pain, nausea, vomiting, and diarrhea), with specificity ranging from 91.73% to 98.43%.

The vaccination status of the participants revealed low vaccination coverage against seasonal influenza, with only 8.33% of influenza-positive cases and 7.87% of influenza-negative cases reporting having been vaccinated against seasonal flu at any point in the past. Sensitivity for this factor was very low (8.33%), and while the specificity was high (92.13%), the positive likelihood ratio (LR+) was minimal, indicating poor utility in distinguishing influenza-positive from influenza-negative cases. Similar results were observed for those vaccinated against influenza in the previous or current year, with sensitivity remaining low and specificity remaining high (above 94%).

The analysis of chronic diseases showed that hypertension was the most common condition among both groups, with 25% of influenza-positive patients and 29.53% of influenza-negative patients reporting a history of hypertension. However, the diagnostic performance of hypertension as a predictor for influenza was relatively weak, with a sensitivity of 25% and specificity of 70.47%, yielding a likelihood ratio (LR+) of

0.85. Other chronic conditions, such as myocardial infarction, stroke, and cardiac insufficiency, were present in smaller proportions but exhibited high specificity (over 98%).
Social and environmental factors, including contact with individuals exhibiting flu-like symptoms in the seven days preceding testing, had higher sensitivity (79.17%) but lower specificity (40.94%).
Smoking, alcohol consumption, and transportation behaviors (e.g., use of buses or taxis) showed moderate diagnostic value, with smoking and alcohol consumption demonstrating relatively high specificity and the ability to rule out influenza in certain contexts.
Lastly, the timing of inclusion in the study revealed slightly different results between the two time periods, with a small drop in accuracy from 0.536 (period 1.12.22–14.2.2023) to 0.464 (period 15.2.2023–30.4.2023).

Patients aged 65 years and older

There numerous symptoms/signs had good balance between sensitivity and specificity such as fever (≥ 38 °C), sudden onset of symptoms, myalgia, malaise, chills with accuracy values ranging from 0.830 to 0.913. Gastrointestinal symptoms, including nausea, vomiting, and diarrhea, demonstrated high specificity, particularly vomiting and diarrhea, which had specificity values of 99.22% and 98.45%, respectively. However, sensitivity of gastrointestinal symptoms/signs had values under 29%, and especially was low (14.29%) for nausea. Shortness of breath showed a sensitivity of 42.86% and specificity of 89.53%, with an LR+ of 4.10 and accuracy of 0.883. Clinical signs of pneumonia had similar performance with an LR+ of 5.27 and an accuracy of 0.906.
Regarding of information about vaccination against seasonal flu ever before, the sensitivity was low (28.57%), specificity was moderate (67.05%), and accuracy was 0.660. Vaccination against the flu last year and vaccination against the flu this year showed similar trends, with sensitivity of 28.57% and specificity ranging from 72.48% to 72.87%, and accuracy with 0.713 and 0.717, respectively.
Hypertension was present in all influenza-positive cases (100%) and in 89.53% of influenza-negative cases, making it a strong indicator of chronic disease, but due to low specificity (10.47%) accuracy was low (0.128). Myocardial infarction and asthma showed high specificity (90.31% and 89.92%, respectively) but sensitivity was 0%. Cardiac insufficiency and angina pectoris had good specificity (94.57% and 85.27%), with cardiac insufficiency showing a higher LR+ (2.63) and accuracy (0.925), indicating it may be a more reliable diagnostic marker than angina pectoris. Diabetes mellitus type 1 and diabetes mellitus type 2 had high specificity and low sensitivity, with diabetes type 1 showing an accuracy of 0.977, while type 2 had lower accuracy (0.762).
The information that a patient lives in a household with children aged 7 to 14 years demonstrated that they significantly more common in influenza-positive cases (14.29%) compared to influenza-negative ones (3.10%), with a high specificity of 96.90% and high accuracy (0.947).
Smoking was associated with a positive case rate of 42.86% in influenza-positive cases, with an accuracy of 0.891.
Transportation behaviors (e.g., use of buses or taxis) demonstrated low predictive value for laboratory-confirmed influenza with accuracy of 0.302 and 0.245.
Compared the later period (15.2.2023–30.4.2023) of inclusion in the study, influenza-positive cases clustered more heavily in the earlier part of the surveillance period (1.12.22 - 14.2.2023) showing sensitivity of 100%, specificity of 50.78% and accuracy of 0.521 (Table S1).

Table S1. Performance of variables related to influenza by age categories during 2022/23 influenza season

**Characteristics**		**15-29 (n=112)**									**30-64 (n=302)**									**≥ 65 (n=265)**								
		Influenza positive (n=28)		Influenza negative (n=84)		Se % (95% CI)	Sp % (95% CI)	LR+ (95% CI)	LR- (95% CI)	Accuracy (95% CI)	Influenza positive (n=48)		Influenza negative (n=254)		Se % (95% CI)	Sp % (95% CI)	LR+ (95% CI)	LR- (95% CI)	Accuracy (95% CI)	Influenza positive (n=7)		Influenza negative (n=258)		Se % (95% CI)	Sp % (95% CI)	LR+ (95% CI)	LR- (95% CI)	Accuracy (95% CI)
		n	%	n	%						n	%	n	%						n	%	n	%					
**Symptoms and signs***	Fever (≥ 38 °C)**	27	96.43	45	53.57	96.43 (81.65-99.91)	46.43 (35.47-57.65)	1.80 (1.46-2.22)	0.08 (0.01-0.53)	0.589 (0.515-0.606)	41	85.42	62	24.41	85.42 (72.24-93.93)	75.59 (69.83-80.74)	3.50 (2.74-4.48)	0.19 (0.10-0.38)	0.772 (0.732-0.797)	5	71.43	29	11.24	71.43 (29.04-96.33)	88.76 (84.26-92.34)	6.35 (3.56-11.36)	0.32 (0.10-1.04)	0.883 (0.862-0.895)
	Cough	26	92.86	57	67.86	92.86 (76.50-99.12)	32.14 (22.36-43.22)	1.37 (1.14-1.64)	0.22 (0.06-0.88)	0.473 (0.396-0.503)	40	83.33	152	59.84	83.33 (69.78-92.52)	40.16 (34.08-46.47)	1.39 (1.18-1.64)	0.42 (0.22-0.80)	0.470 (0.429-00.497)	5	71.43	104	40.31	71.43 (29.04-96.33)	59.69 (53.43-65.73)	1.77 (1.08-2.90)	0.48 (0.15-1.55)	0.600 (0.578-0.612)
	Sudden onset of symptoms	25	89.29	59	70.24	89.29 (71.77-97.73)	29.76 (20.27-40.73)	1.27 (1.05-1.54)	0.36 (0.12-1.10)	0.446 (0.367-0.486)	35	72.92	84	33.07	72.92 (58.15-84.72)	66.93 (60.77-72.68)	2.20 (1.72-2.82)	0.40 (0.25-0.65)	0.679 (0.635-0.713)	5	71.43	43	16.67	71.43 (29.04-96.33)	83.33 (78.21-87.67)	4.29 (2.49-7.37)	0.34 (0.11-1.11)	0.830 (0.809-0.843)
	Headache	26	92.86	54	64.29	92.86 (76.50-99.12)	35.71 (25.55-46.92)	1.44 (1.19-1.75)	0.20 (0.05-0.78)	0.500 (0.422-0.529)	23	47.92	80	31.50	47.92 (33.29-62.81)	68.50 (62.40-74.17)	1.52 (1.08-2.15)	0.76 (0.57-1.01)	0.652 (0.610-0.695)	2	28.57	39	15.12	28.57 (3.67-70.96)	84.88 (79.92-89.02)	1.89 (0.57-6.32)	0.84 (0.53-1.35)	0.834 (0.822-0.855)
	Dizziness	10	35.71	26	30.95	35.71 (18.64-55.93)	69.05 (58.02-78.69)	1.15 (0.64-2.08)	0.93 (0.68-1.27)	0.607 (0.531-0.693)	13	27.08	26	10.24	27.08 (15.28-41.85)	89.76 (85.36-93.20)	2.65 (1.47-4.77)	0.81 (0.68-0.97)	0.798 (0.765-0.836)	0	0.00	29	11.24	0.00 (0.00-40.96)	88.76 (84.26-92.34)	NA	1.13 (1.08-1.18)	0.864 (0.864-0.887)
	Sore throat	23	82.14	64	76.19	82.14 (63.11-93.94)	23.81 (15.19-34.35)	1.08 (0.87-1.33)	0.75 (0.31-1.81)	0.384 (0.303-0.438)	38	79.17	232	91.34	79.17 (65.01-89.53)	8.66 (5.51-12.82)	0.87 (0.75-1.01)	2.41 (1.22-4.75)	0.199 (0.163-0.228)	4	57.14	241	93.41	57.14 (18.41-90.10)	6.59 (3.88-10.34)	0.61 (0.32-1.16)	6.50 (2.46-17.17)	0.079 (0.060-0.096)
	Nasal congestion	28	100.00	70	83.33	100.00 (87.66-100.00)	16.67 (9.42-26.38)	1.20 (1.09-1.32)	NA	0.375 (0.313-0.375)	38	79.17	231	90.94	79.17 (65.01-89.53)	9.06 (5.83-13.28)	0.87 (0.75-1.01)	2.30 (1.17-4.52)	0.202 (0.166-0.232)	6	85.71	238	92.25	85.71 (42.13-99.64)	7.75 (4.80-11.72)	0.93 (0.69-1.26)	1.84 (0.29-11.87)	0.098 (0.076-0.105)
	Myalgia	23	82.14	42	50.00	82.14 (63.11-93.94)	50.00 (38.89-61.11)	1.64 (1.25-2.16)	0.36 (0.16-0.81)	0.580 (0.494-0.635)	32	66.67	64	25.20	66.67 (51.59-79.60)	74.80 (69.00-80.02)	2.65 (1.98-3.54)	0.45 (0.30-0.67)	0.735 (0.691-0.772)	5	71.43	32	12.40	71.43 (29.04-96.33)	87.60 (82.94-91.36)	5.76 (3.26-10.18)	0.33 (0.10-1.05)	0.872 (0.850-0.884)
	Malaise	27	96.43	56	66.67	96.43 (81.65-99.91)	33.33 (23.42-44.46)	1.45 (1.22-1.71)	0.11 (0.02-0.75)	0.491 (0.418-0.508)	37	77.08	87	34.25	77.08 (62.69-87.97)	65.75 (59.56-71.57)	2.25 (1.79-2.83)	0.35 (0.21-0.59)	0.675 (0.632-0.708)	6	85.71	32	12.40	85.71 (42.13-99.64)	87.60 (82.94-91.36)	6.91 (4.44-10.77)	0.16 (0.03-1.00)	0.875 (0.853-0.883)
	Chills	22	78.57	36	42.86	78.57 (59.05-91.70)	57.14 (45.88-67.89)	1.83 (1.34-2.51)	0.38 (0.18-0.78)	0.625 (0.537-0.685)	28	58.33	59	23.23	58.33 (43.21-72.39)	76.77 (71.08-81.82)	2.51 (1.81-3.48)	0.54 (0.39-0.76)	0.738 (0.695-0.778)	4	57.14	20	7.75	57.14 (18.41-90.10)	92.25 (88.28-95.20)	7.37 (3.42-15.88)	0.46 (0.20-1.09)	0.913 (0.894-0.929)
	Loss of appetite	13	46.43	30	35.71	46.43 (27.51-66.13)	64.29 (53.08-74.45)	1.30 (0.80-2.12)	0.83 (0.57-1.22)	0.598 (0.515-0.683)	5	10.42	21	8.27	10.42 (3.47-22.66)	91.73 (87.64-94.81)	1.26 (0.50-3.18)	0.98 (0.88-1.08)	0.788 (0.768-0.821)	2	28.57	10	3.88	28.57 (3.67-70.96)	96.12 (92.99-98.13)	7.37 (1.97-27.58)	0.74 (0.46-1.19)	0.943 (0.931-0.964)
	Abdominal pain	3	10.71	22	26.19	10.71 (2.27-28.23)	73.81 (63.07-82.80)	0.41 (0.13-1.26)	1.21 (1.01-1.45)	0.580 (0.541-0.658)	3	6.25	13	5.12	6.25 (1.31-17.20)	94.88 (91.41-97.25)	1.22 (0.36-4.12)	0.99 (0.91-1.07)	0.808 (0.793-0.836)	2	28.57	8	3.10	28.57 (3.67-70.96)	96.90 (93.98-98.65)	9.21 (2.38-35.74)	0.74 (0.46-1.18)	0.951 (0.939-0.971)
	Nausea	3	10.71	18	21.43	10.71 (2.27-28.23)	78.57 (68.26-86.78)	0.50 (0.16-1.57)	1.14 (0.96-1.35)	0.616 (0.577-0.692)	1	2.08	13	5.12	2.08 (0.05-11.07)	94.88 (91.41-97.25)	0.41 (0.05-3.04)	1.03 (0.98-1.09)	0.801 (0.795-0.827)	1	14.29	7	2.71	14.29 (0.36-57.87)	97.29 (94.49-98.90)	5.27 (0.74-37.24)	0.88 (0.65-1.19)	0.951 (0.944-0.971)
	Vomiting	3	10.71	10	11.90	10.71 (2.27-28.23)	88.10 (79.19-94.14)	0.90 (0.27-3.04)	1.01 (0.87-1.18)	0.688 (0.649-0.754)	2	4.17	5	1.97	4.17 (0.51-14.25)	98.03 (95.47-99.36)	2.12 (0.42-10.60)	0.98 (0.92-1.04)	0.831 (0.820-0.850)	2	28.57	2	0.78	28.57 (3.67-70.96)	99.22 (97.23-99.91)	36.86 (6.03-225.33)	0.72 (0.45-1.15)	0.974 (0.961-0.986)
	Diarrhea	2	7.14	17	20.24	7.14 (0.88-23.50)	79.76 (69.59-87.75)	0.35 (0.09-1.43)	1.16 (1.00-1.35)	0.616 (0.587-0.689)	0	0.00	4	1.57	0.00 (0.00-7.40)	98.43 (96.02-99.57)	NA	1.02 (1.00-1.03)	0.828 (0.828-0.844)	2	28.57	4	1.55	28.57 (3.67-70.96)	98.45 (96.08-99.58)	18.43 (4.02-84.46)	0.73 (0.45-1.16)	0.966 (0.954-0.983)
	Shortness of breath	3	10.71	5	5.95	10.71 (2.27-28.23)	94.05 (86.65-98.04)	1.80 (0.46-7.06)	0.95 (0.83-1.09)	0.732 (0.693-0.783)	9	18.75	36	14.17	18.75 (8.95-32.63)	85.83 (80.92-89.87)	1.32 (0.68-2.56)	0.95 (0.82-1.09)	0.752 (0.723-0.790)	3	42.86	27	10.47	42.86 (9.90-81.59)	89.53 (85.14-92.99)	4.10 (1.62-10.35)	0.64 (0.34-1.21)	0.883 (0.867-0.902)

	Clinical signs of pneumonia (auscultatory)	0	0.00	1	1.19	0.00 (0.00-12.34)	98.81 (93.54-99.97)	NA	1.01 (0.99-1.04)	0.741 (0.741-0.758)	5	10.42	20	7.87	10.42 (3.47-22.66)	92.13 (88.10-95.12)	1.32 (0.52-3.35)	0.97 (0.88-1.08)	0.791 (0.771-0.824)	3	42.86	21	8.14	42.86 (9.90-81.59)	91.86 (87.83-94.89)	5.27 (2.04-13.60)	0.62 (0.33-1.18)	0.906 (0.889-0.925)
**Vaccination status**	Vaccinated against seasonal flu ever before	0	0.00	1	1.19	0.00 (0.00-12.34)	98.81 (93.54-99.97)	NA	1.01 (0.99-1.04)	0.741 (0.741-0.758)	4	8.33	20	7.87	8.33 (2.32-19.98)	92.13 (88.10-95.12)	1.06 (0.38-2.96)	1.00 (0.91-1.09)	0.788 (0.770-0.820)	2	28.57	85	32.95	28.57 (3.67-70.96)	67.05 (60.95-72.76)	0.87 (0.27-2.83)	1.07 (0.66-1.72)	0.660 (0.648-0.682)
	Vaccinated against the flu last year	0	0.00	0	0.00	NA	NA	NA	NA	NA	2	4.17	13	5.12	4.17 (0.51-14.25)	94.88 (91.41-97.25)	0.81 (0.19-3.49)	1.01 (0.95-1.08)	0.805 (0.794-0.831)	2	28.57	70	27.13	28.57 (3.67-70.96)	72.87 (67.01-78.20)	1.05 (0.32-3.46)	0.98 (0.61-1.58)	0.717 (0.705-0.739)
	Vaccinated against the flu this year	0	0.00	0	0.00	NA	NA	NA	NA	NA	2	4.17	13	5.12	4.17 (0.51-14.25)	94.88 (91.41-97.25)	0.81 (0.19-3.49)	1.01 (0.95-1.08)	0.805 (0.794-0.831)	2	28.57	71	27.52	28.57 (3.67-70.96)	72.48 (66.60-77.84)	1.04 (0.32-3.41)	0.99 (0.61-1.58)	0.713 (0.701-0.735)
	Vaccinated against COVID-19 in a timely manner	4	14.29	18	21.43	14.29 (4.03-32.67)	78.57 (68.26-86.78)	0.67 (0.25-1.80)	1.09 (0.90-1.32)	0.625 (0.578-0.703)	26	54.17	157	61.81	54.17 (39.17-68.63)	38.19 (32.19-44.47)	0.88 (0.66-1.16)	1.20 (0.85-1.69)	0.407 (0.364-0.450)	5	71.43	204	79.07	71.43 (29.04-96.33)	20.93 (16.13-26.41)	0.90 (0.56-1.45)	1.37 (0.41-4.51)	0.223 (0.201-0.235)
**Chronic disease*** **	Hypertension	0	0.00	0	0.00	NA	NA	NA	NA	NA	12	25.00	75	29.53	25.00 (13.64-39.60)	70.47 (64.45-76.01)	0.85 (0.50-1.43)	1.06 (0.89-1.28)	0.632 (0.599-0.675)	7	100.00	231	89.53	100.00 (59.04-100.00)	10.47 (7.01-14.86)	1.12 (1.07-1.16)	NA	0.128 (0.106-0.128)
	Myocardial infarction	0	0.00	0	0.00	NA	NA	NA	NA	NA	1	2.08	5	1.97	2.08 (0.05-11.07)	98.03 (95.47-99.36)	1.06 (0.13-8.86)	1.00 (0.96-1.04)	0.828 (0.822-0.846)	0	0.00	25	9.69	0.00 (0.00-40.96)	90.31 (86.03-93.63)	NA	1.11 (1.06-1.15)	0.879 (0.879-0.902)
	Cardiac insuficience	0	0.00	0	0.00	NA	NA	NA	NA	NA	1	2.08	1	0.39	2.08 (0.05-11.07)	99.61 (97.83-99.99)	5.29 (0.34-83.16)	0.98 (0.94-1.03)	0.841 (0.835-0.847)	1	14.29	14	5.43	14.29 (0.36-57.87)	94.57 (91.06-97.00)	2.63 (0.40-17.34)	0.91 (0.67-1.23)	0.925 (0.917-0.946)
	Angina pectoris	0	0.00	0	0.00	NA	NA	NA	NA	NA	0	0.00	4	1.57	0.00 (0.00-7.40)	98.43 (96.02-99.57)	NA	1.02 (1.00-1.03)	0.828 (0.828-0.844)	2	28.57	38	14.73	28.57 (3.67-70.96)	85.27 (80.35-89.36)	1.94 (0.58-6.49)	0.84 (0.52-1.34)	0.838 (0.825-0.859)
	Arrhythmia	0	0.00	0	0.00	NA	NA	NA	NA	NA	1	2.08	11	4.33	2.08 (0.05-11.07)	95.67 (92.38-97.82)	0.48 (0.06-3.64)	1.02 (0.97-1.07)	0.808 (0.802-0.832)	1	14.29	39	15.12	14.29 (0.36-57.87)	84.88 (79.92-89.02)	0.95 (0.15-5.94)	1.01 (0.74-1.37)	0.830 (0.823-0.853)
	Stroke	0	0.00	0	0.00	NA	NA	NA	NA	NA	1	2.08	1	0.39	2.08 (0.05-11.07)	99.61 (97.83-99.99)	5.29 (0.34-83.16)	0.98 (0.94-1.03)	0.841 (0.835-0.847)	1	14.29	16	6.20	14.29 (0.36-57.87)	93.80 (90.12-96.41)	2.30 (0.35-15.03)	0.91 (0.67-1.24)	0.917 (0.910-0.939)
	Asthma	2	7.14	3	3.57	7.14 (0.88-23.50)	96.43 (89.92-99.26)	2.00 (0.35-11.36)	0.96 (0.86-1.08)	0.741 (0.712-0.779)	2	4.17	24	9.45	4.17 (0.51-14.25)	90.55 (86.27-93.85)	0.44 (0.11-1.80)	1.06 (0.99-1.14)	0.768 (0.757-0.798)	0	0.00	26	10.08	0.00 (0.00-40.96)	89.92 (85.58-93.31)	NA	1.11 (1.07-1.16)	0.875 (0.875-0.898)
	Diabetes mellitus type 1	0	0.00	2	2.38	0.00 (0.00-12.34)	97.62 (91.66-99.71)	NA	1.02 (0.99-1.06)	0.732 (0.732-0.761)	0	0.00	1	0.39	0.00 (0.00-7.40)	99.61 (97.83-99.99)	NA	1.00 (1.00-1.01)	0.838 (0.838-0.844)	1	14.29	0	0.00	14.29 (0.36-57.87)	100.00 (98.58-100.00)	NA	0.86 (0.63-1.16)	0.977 (0.970-0.977)
	Diabetes mellitus type 2	0	0.00	0	0.00	NA	NA	NA	NA	NA	3	6.25	15	5.91	6.25 (1.31-17.20)	94.09 (90.45-96.66)	1.06 (0.32-3.52)	1.00 (0.92-1.08)	0.801 (0.787-0.830)	1	14.29	57	22.09	14.29 (0.36-57.87)	77.91 (72.34-82.82)	0.65 (0.10-4.03)	1.10 (0.81-1.50)	0.762 (0.755-0.785)
	Obesity	0	0.00	1	1.19	0.00 (0.00-12.34)	98.81 (93.54-99.97)	NA	1.01 (0.99-1.04)	0.741 (0.741-0.758)	3	6.25	25	9.84	6.25 (1.31-17.20)	90.16 (85.81-93.53)	0.63 (0.20-2.02)	1.04 (0.96-1.13)	0.768 (0.754-0.800)	1	14.29	22	8.53	14.29 (0.36-57.87)	91.47 (87.37-94.58)	1.68 (0.26-10.74)	0.94 (0.69-1.27)	0.894 (0.887-0.917)
	Other	2	7.14	9	10.71	7.14 (0.88-23.50)	89.29 (80.63-94.98)	0.67 (0.15-2.90)	1.04 (0.92-1.18)	0.688 (0.658-0.750)	19	39.58	110	43.31	39.58 (25.77-54.73)	56.69 (50.35-62.87)	0.91 (0.63-1.33)	1.07 (0.83-1.37)	0.540 (0.499-0.584)	3	42.86	158	61.24	42.86 (9.90-81.59)	38.76 (32.78-45.00)	0.70 (0.30-1.66)	1.47 (0.76-2.85)	0.389 (0.372-0.408)
	Without chronic diseases	24	85.71	70	83.33	85.71 (67.33-95.97)	16.67 (9.42-26.38)	1.03 (0.86-1.23)	0.86 (0.31-2.39)	0.339 (0.265-0.386)	23	47.92	94	37.01	47.92 (33.29-62.81)	62.99 (56.73-68.94)	1.29 (0.93-1.81)	0.83 (0.62-1.10)	0.606 (0.563-0.649)	0	0.00	5	1.94	0.00 (0.00-40.96)	98.06 (95.54-99.3)	NA	1.02 (1.00-1.04)	0.955 (0.955-0.973)
**Children aged 7 to 14 in the patient's family**		7	25.00	16	19.05	25.00 (10.69-44.87)	80.95 (70.92-88.70)	1.31 (0.60-2.86)	0.93 (0.73-1.18)	0.670 (0.605-0.749)	17	35.42	72	28.35	35.42 (22.16-50.54)	71.65 (65.68-77.11)	1.25 (0.81-1.92)	0.90 (0.72-1.13)	0.606 (0.563-0.649)	1	14.29	8	3.10	14.29 (0.36-57.87)	96.90 (93.98-98.65)	4.61 (0.66-32.01)	0.88 (0.65-1.20)	0.947 (0.940-0.968)
**Children aged 15 to 18 in the patient's family**		1	3.57	7	8.33	3.57 (0.09-18.35)	91.67 (83.58-96.58)	0.43 (0.06-3.33)	1.05 (0.96-1.16)	0.696 (0.680-0.752)	7	14.58	36	14.17	14.58 (6.07-27.76)	85.83 (80.92-89.87)	1.03 (0.49-2.18)	1.00 (0.88-1.13)	0.745 (0.720-0.782)	0	0.00	6	2.33	0.00 (0.00-40.96)	97.67 (95.01-99.14)	NA	1.02 (1.00-1.04)	0.951 (0.951-0.970)

**Contact with someone who had flu-like symptoms seven days before testing**		24	85.71	55	65.48	85.71 (67.33-95.97)	34.52 (24.48-45.69)	1.31 (1.05-1.63)	0.41 (0.16-1.07)	0.473 (0.391-0.521)	38	79.17	150	59.06	79.17 (65.01-89.53)	40.94 (34.84-47.27)	1.34 (1.12-1.60)	0.51 (0.29-0.90)	0.470 (0.428-0.501)	5	71.43	66	25.58	71.43 (29.04-96.33)	74.42 (68.64-79.63)	2.79 (1.67-4.66)	0.38 (0.12-1.24)	0.743 (0.722-0.756)
**Smoking**		5	17.86	18	21.43	17.86 (6.06-36.89)	78.57 (68.26-86.78)	0.83 (0.34-2.04)	1.05 (0.85-1.28)	0.634 (0.580-0.713)	12	25.00	71	27.95	25.00 (13.64-39.60)	72.05 (66.10-77.48)	0.89 (0.53-1.52)	1.04 (0.87-1.25)	0.646 (0.612-0.688)	3	42.86	25	9.69	42.86 (9.90-81.59)	90.31 (86.03-93.63)	4.42 (1.74-11.24)	0.63 (0.33-1.20)	0.891 (0.874-0.910)
**Alcohol consumption**		6	21.43	8	9.52	21.43 (8.30-40.95)	90.48 (82.09-95.80)	2.25 (0.85-5.93)	0.87 (0.71-1.07)	0.732 (0.674-0.797)	2	4.17	5	1.97	4.17 (0.51-14.25)	98.03 (95.47-99.36)	2.12 (0.42-10.60)	0.98 (0.92-1.04)	0.831 (0.820-0.850)	0	0.00	2	0.78	0.00 (0.00-40.96)	99.22 (97.23-99.91)	NA	1.01 (1.00-1.02)	0.966 (0.966-0.978)
**Use of buses for transportation purposes**		24	85.71	71	84.52	85.71 (67.33-95.97)	15.48 (8.51-25.01)	1.01 (0.85-1.21)	0.92 (0.33-2.60)	0.330 (0.257-0.377)	30	62.50	183	72.05	62.50 (47.35-76.05)	27.95 (22.52-33.90)	0.87 (0.69-1.09)	1.34 (0.89-2.03)	0.334 (0.291-0.374)	6	85.71	184	71.32	85.71 (42.13-99.64)	28.68 (23.24-34.62)	1.20 (0.88-1.64)	0.50 (0.08-3.09)	0.302 (0.279-0.309)
**Use of TAXI for transportation purposes**		17	60.71	52	61.90	60.71 (40.58-78.50)	38.10 (27.71-49.34)	0.98 (0.70-1.38)	1.03 (0.60-1.76)	0.438 (0.350-0.517)	42	87.50	218	85.83	87.50 (74.75-95.27)	14.17 (10.13-19.08)	1.02 (0.91-1.15)	0.88 (0.39-1.98)	0.258 (0.222-0.281)	4	57.14	197	76.36	57.14 (18.41-90.10)	23.64 (18.59-29.31)	0.75 (0.39-1.43)	1.81 (0.75-4.38)	0.245 (0.226-0.262)
**Period of inclusion in the study**	1.12.22-14.2.2023.	20	71.43	36	42.86	71.43 (51.33-86.78)	57.14 (45.88-67.89)	1.67 (1.19-2.34)	0.50 (0.27-0.92)	0.607 (0.518-0.677)	25	52.08	117	46.06	52.08 (37.19-66.71)	53.94 (47.60-60.19)	1.13 (0.84-1.53)	0.89 (0.65-1.22)	0.536 (0.493-0.579)	7	100.00	127	49.22	100.00 (59.04-100.00)	50.78 (44.50-57.03)	2.03 (1.79-2.30)	NA	0.521 (0.498-0.521)
	15.2.2023-30.4.2023.	8	28.57	48	57.14	28.57 (13.22-48.67)	42.86 (32.11-54.12)	0.50 (0.27-0.92)	1.67 (1.19-2.34)	0.393 (0.323-0.482)	23	47.92	137	53.94	47.92 (33.29-62.81)	46.06 (39.81-52.40)	0.89 (0.65-1.22)	1.13 (0.84-1.53)	0.464 (0.421-0.507)	0	0.00	131	50.78	0.00 (0.00-40.96)	49.22 (42.97-55.50)	NA	2.03 (1.79-2.30)	0.479 (0.479-0.502)

*one patient could have one or more symptoms, simultaneously; **including feverishness; ***one patient could have one or more comorbidities, simultaneously; Se: sensitivitiy; Sp: specificity; LR+: positive likelihood ratio; LR-: negative likelihood ratio.
