# Supplementary material for: Diagnostic Significance of Influenza Symptoms and Signs, and Their Variation by Type/Subtype, in Outpatients Aged ≥ 15 Years: Novi Sad, Serbia
Source: Viruses. 2025 Feb 16;17(2):272. doi: 10.3390/v17020272 (PMC11860240; doi:10.3390/v17020272)
Supplement: Supplementary file 1 [file viruses-17-00272-s001.zip › Suppl. S2.pdf]

Section S2

Performance of variables related to influenza by age categories during 2023/24 influenza season

Patients aged 15-29 years

Cough, fever (≥ 38°C), myalgia, and malaise**,** all exhibited relatively high sensitivity (100.00%, 92.31%, 84.62%, and 84.62%, respectively), but their specificity was lower (between 11.96% and 59.78%). Among all symptoms/signs, the highest specificity was detected for the gastrointestinal symptoms/signs and ranging from 93.48% (loss of appetite, diarrhea and nausea) to 96.74% (vomiting).

Vaccination rates in this age group were negligible.

Regarding chronic diseases, only a small proportion of individuals in this age group had such conditions. Consequently, the absence of chronic diseases showed high sensitivity (92.31%), but due to low specificity (15.22%), the overall accuracy was only 0.248.

The information that a patient lives in a household with children aged 7 to 14 years demonstrated a low sensitivity (7.69%) and a specificity of 96.74%. Contact during seven days before testing with someone who had flu-like symptoms had sensitivity of 69.23%, but very low specificity (9.78%), resulting in low accuracy (0.171).

Smoking demonstrated moderate sensitivity (15.38%), high specificity (92.39%), and an accuracy of 0.829. The use of buses for transportation exhibited high sensitivity (76.92%) but low specificity (9.78%). In contrast, the use of taxi for transportation showed lower sensitivity (53.85%) but higher specificity (19.57%) when compared to bus usage.

Considering the period 1.12.23–14.2.2024 in order to influenza detection, there was a high sensitivity (92.31%) and moderate specificity (46.74%), resulting in moderate accuracy (0.524). In other hand, the period 15.2.2024–30.4.2024 showed evidently lower sensitivity (7.69%) and higher specificity (53.26%) resulting accuracy of 0.476.

Patients aged 30-64 years

In patients aged 30–64 years, there was low sensitivity observed by symptoms/signs related to influenza. The maximum value of sensitivity (100%) was detected for fever (≥ 38°C), cough, headache and malaise, but due to their low specificities (53.41%, 14.77%, 59.09%, and 38.92%, respectively), the accuracy had moderate or low values (0.549, 0.176, 0.604, and 0.409, respectively).

Similar to the 15-29 age group, the highest specificity values for patients aged 30-64 years were noted for gastrointestinal symptoms and signs (loss of appetite, abdominal pain, nausea, vomiting, and diarrhea), where specificity ranging from 83.24% to 98.30%

The vaccination status of the participants revealed low vaccination coverage against seasonal influenza, with only 8.33% of influenza-positive cases and 6.25% of influenza-negative cases reporting having been vaccinated against seasonal flu at any point in the past. The specificity for this factor was high (93.75%), but sensitivity was very low (8.33%). Similar results were observed for those vaccinated against influenza in the previous or current year, with sensitivity remaining low and specificity remaining high (above 94%).

The analysis of chronic diseases showed that hypertension was the most common condition among both groups, with 33.33% of influenza-positive patients and 22.44% of influenza-negative patients reporting a history of hypertension. However, the diagnostic performance of hypertension as a predictor for influenza was relatively weak, with a sensitivity of 33.33% and specificity of 77.56%. Other chronic conditions, such as myocardial infarction, cardiac insufficiency, angina pectoris, and stroke were present in smaller proportions but exhibited high specificity (above 99%).

Social and environmental factors, including contact with individuals exhibiting flu-like symptoms in the seven days preceding testing, demonstrated a sensitivity of 100% but lower specificity (22.44%). In contrast, living in a household with children aged 7–14 or 15–18 years was associated with a sensitivity of 8.33% (both variables) and a specificity of 87.22% (for children aged 7–14), and 88.35% (for children aged 15-18 years).

Transportation behaviors (e.g., the use of buses or taxis) demonstrated moderate diagnostic value. The use of buses showed a sensitivity of 83.33% and a specificity of 17.05%, while the use of taxis exhibited a sensitivity of 91.67% and a specificity of 15.91%. Both behaviors resulted in low accuracy values (0.192 and 0.184, respectively).

Sensitivity during the first period (1.12.23–14.2.2024) was significantly higher (75%) compared to the second period (25%), whereas specificity was lower (30.68%) than in the second period (15.2.2024–30.4.2024) when was 69.32%. The overall accuracy for the first period was 0.321, while for the second period was 0.679.

Patients aged 65 years and older

The presence of fever (≥ 38 °C) and sudden onset of symptoms demonstrated a favorable trade-off between sensitivity and specificity. Sensitivity for fever was 75%, and specificity was 80.14%, yielding an accuracy of 0.801. In contrast, sensitivity for sudden onset of symptoms was 50%, and specificity was 80.85%, yielding an accuracy of 0.804. Other symptoms/signs demonstrated low performance in order to detect influenza.

Gastrointestinal symptoms, including loss of appetite, abdominal pain, nausea, vomiting, and diarrhea, demonstrated high specificity ranging from 92.55% to 99.29%.

Regarding of information about vaccination against seasonal flu ever before, the sensitivity was 50%, specificity was high (81.21%), and accuracy was 0.808. Vaccination against the flu last year and vaccination against the flu this year showed similar trends, with sensitivity of 50% and specificity ranging from 82.27% to 83.33%.

Hypertension was present in 75% influenza-positive cases and in 91.13% of influenza-negative cases, making it a strong indicator of chronic disease, but with a very low accuracy (0.098).

There were no patients with laboratory-confirmed influenza who lived in households with children aged 7 to 18 years, nor were there any smokers or alcohol consumers among them.

Contact with someone who had flu-like symptoms seven days before testing had maximum sensitivity (100%), but due to low specificity (21.63%) the accuracy was only 0.227.

Transportation behaviors (e.g., use of buses or taxis) demonstrated low predictive value for laboratory-confirmed influenza, i.e. accuracy were 0.098 and 0.126, respectively.

Months of inclusion in the surveillance showed an interesting temporal pattern, when all laboratory confirmed influenza cases were detected in the earlier part of the surveillance period (1.12.23 - 14.2.2024), showing sensitivity of 100% (Table S2).

Table S2. Performance of variables related to influenza by age categories during 2023/24 influenza season

Characteristics		15-29 (n=105)									30-64 (n=364)									≥ 65 (n=286)								
		Influenza positive (n=13)		Influenza negative (n=92)		Se % (95% CI)	Sp % (95% CI)	LR+ (95% CI)	LR- (95% CI)	Accuracy (95% CI)	Influenza positive (n=12)		Influenza negative (n=352)		Se % (95% CI)	Sp % (95% CI)	LR+ (95% CI)	LR- (95% CI)	Accuracy (95% CI)	Influenza positive (n=4)		Influenza negative (n=282)		Se % (95% CI)	Sp % (95% CI)	LR+ (95% CI)	LR- (95% CI)	Accuracy (95% CI)
		n	%	n	%						n	%	n	%						n	%	n	%					
Symptoms and signs*	Fever (≥ 38 °C)**	12	92.31	54	58.70	92.31 (63.97-99.81)	41.30 (31.13-52.05)	1.57 (1.25-1.98)	0.19 (0.03-1.24)	0.476 (0.406-0.494)	12	100.00	164	46.59	100.00 (73.54-100.00)	53.41 (48.05-58.71)	2.15 (1.92-2.40)	NA	0.549 (0.530-0.549)	3	75.00	56	19.86	75.00 (19.41-99.37)	80.14 (75.00-84.64)	3.78 (2.05-6.97)	0.31 (0.06-1.70)	0.801 (0.786-0.807)
	Cough	13	100.00	81	88.04	100.00 (75.29-100.00)	11.96 (6.12-20.39)	1.14 (1.05-1.22)	NA	0.229 (0.169-0.229)	12	100.00	300	85.23	100.00 (73.54-100.00)	14.77 (11.23-18.92)	1.17 (1.12-1.23)	NA	0.176 (0.157-0.176)	4	100.00	244	86.52	100.00 (39.76-100.00)	13.48 (9.72-18.02)	1.16 (1.10-1.21)	NA	0.147 (0.130-0.147)
	Sudden onset of symptoms	7	53.85	39	42.39	53.85 (25.13-80.78)	57.61 (46.86-67.85)	1.27 (0.73-2.22)	0.80 (0.43-1.48)	0.571 (0.505-0.633)	11	91.67	155	44.03	91.67 (61.52-99.79)	55.97 (50.61-61.22)	2.08 (1.69-2.56)	0.15 (0.02-0.97)	0.571 (0.551-0.577)	2	50.00	54	19.15	50.00 (6.76-93.24)	80.85 (75.77-85.27)	2.61 (0.95-7.16)	0.62 (0.23-1.65)	0.804 (0.793-0.816)
	Headache	10	76.92	39	42.39	76.92 (46.19-94.96)	57.61 (46.86-67.85)	1.81 (1.24-2.66)	0.40 (0.15-1.10)	0.600 (0.528-0.642)	12	100.00	144	40.91	100.00 (73.54-100.00)	59.09 (53.75-64.27)	2.44 (2.16-2.77)	NA	0.604 (0.585-0.604)	0	0.00	35	12.41	0.00 (0.00-60.24)	87.59 (83.16-91.20)	NA	1.14 (1.09-1.19)	0.864 (0.864-0.880)
	Dizziness	1	7.69	9	9.78	7.69 (0.19-36.03)	90.22 (82.24-95.43)	0.79 (0.11-5.71)	1.02 (0.86-1.21)	0.800 (0.782-0.861)	1	8.33	41	11.65	8.33 (0.21-38.48)	88.35 (84.53-91.51)	0.72 (0.11-4.78)	1.04 (0.87-1.24)	0.857 (0.852-0.877)	0	0.00	21	7.45	0.00 (0.00-60.24)	92.55 (88.84-95.33)	NA	1.08 (1.05-1.12)	0.913 (0.913-0.929)
	Sore throat	8	61.54	55	59.78	61.54 (31.58-86.14)	40.22 (30.12-50.96)	1.03 (0.65-1.63)	0.96 (0.46-1.99)	0.429 (0.359-0.485)	9	75.00	178	50.57	75.00 (42.81-94.51)	49.43 (44.09-54.78)	1.48 (1.05-2.09)	0.51 (0.19-1.36)	0.503 (0.482-0.515)	3	75.00	184	65.25	75.00 (19.41-99.37)	34.75 (29.20-40.62)	1.15 (0.65-2.04)	0.72 (0.13-3.96)	0.353 (0.338-0.360)
	Nasal congestion	7	53.85	81	88.04	53.85 (25.13-80.78)	11.96 (6.12-20.39)	0.61 (0.37-1.02)	3.86 (1.72-8.66)	0.171 (0.110-0.232)	5	41.67	300	85.23	41.67 (15.17-72.33)	14.77 (11.23-18.92)	0.49 (0.25-0.96)	3.95 (2.30-6.78)	0.157 (0.140-0.176)	4	100.00	243	86.17	100.00 (39.76-100.00)	13.83 (10.02-18.42)	1.16 (1.11-1.22)	NA	0.150 (0.134-0.150)
	Myalgia	11	84.62	37	40.22	84.62 (54.55-98.08)	59.78 (49.04-69.88)	2.10 (1.50-2.96)	0.26 (0.07-0.93)	0.629 (0.556-0.660)	4	33.33	181	51.42	33.33 (9.92-65.11)	48.58 (43.25-53.94)	0.65 (0.29-1.45)	1.37 (0.91-2.08)	0.481 (0.466-0.501)	0	0.00	94	33.33	0.00 (0.00-60.24)	66.67 (60.83-72.14)	NA	1.50 (1.38-1.63)	0.657 (0.657-0.674)
	Malaise	11	84.62	47	51.09	84.62 (54.55	48.91 (38.3	1.66 (1.22-2.25)	0.31 (0.09-1.14)	0.533 (0.461-0.565)	12	100.00	215	61.08	100.00 (73.54-100.00)	38.92 (33.8	1.64 (1.51-	NA	0.409 (0.390-0.409)	0	0.00	92	32.62	0.00 (0.00-60.24)	67.38 (61.5	NA	1.48 (1.37-	0.664 (0.664-0.681)

						-98.08)	4-59.56)									0-44.23)	1.78)								7-72.82)		1.61)	
	Chills	4	30.77	16	17.39	30.77 (9.09-61.43)	82.61 (73.30-89.72)	1.77 (0.70-4.48)	0.84 (0.58-1.22)	0.762 (0.712-0.830)	2	16.67	93	26.42	16.67 (2.09-48.41)	73.58 (68.65-78.11)	0.63 (0.18-2.26)	1.13 (0.87-1.47)	0.717 (0.708-0.738)	0	0.00	26	9.22	0.00 (0.00-60.24)	90.78 (86.78-93.89)	NA	1.10 (1.06-1.14)	0.895 (0.895-0.912)
	Loss of appetite	3	23.08	6	6.52	23.08 (5.04-53.81)	93.48 (86.34-97.57)	3.54 (1.01-12.45)	0.82 (0.61-1.11)	0.848 (0.807-0.904)	1	8.33	59	16.76	8.33 (0.21-38.48)	83.24 (78.92-86.99)	0.50 (0.08-3.29)	1.10 (0.92-1.31)	0.808 (0.802-0.828)	0	0.00	21	7.45	0.00 (0.00-60.24)	92.55 (88.84-95.33)	NA	1.08 (1.05-1.12)	0.913 (0.913-0.929)
	Abdominal pain	2	15.38	4	4.35	15.38 (1.92-45.45)	95.65 (89.24-98.80)	3.54 (0.72-17.44)	0.88 (0.70-1.12)	0.857 (0.826-0.904)	0	0.00	28	7.95	0.00 (0.00-26.46)	92.05 (88.71-94.65)	NA	1.09 (1.05-1.12)	0.890 (0.890-0.909)	0	0.00	2	0.71	0.00 (0.00-60.24)	99.29 (97.46-99.91)	NA	1.01 (1.00-1.02)	0.979 (0.979-0.990)
	Nausea	2	15.38	6	6.52	15.38 (1.92-45.45)	93.48 (86.34-97.57)	2.36 (0.53-10.48)	0.91 (0.71-1.15)	0.838 (0.807-0.894)	0	0.00	22	6.25	0.00 (0.00-26.46)	93.75 (90.69-96.04)	NA	1.07 (1.04-1.10)	0.907 (0.907-0.925)	1	25.00	17	6.03	25.00 (0.63-80.59)	93.97 (90.52-96.45)	4.15 (0.71-24.08)	0.80 (0.45-1.41)	0.930 (0.923-0.945)
	Vomiting	1	7.69	3	3.26	7.69 (0.19-36.03)	96.74 (90.77-99.32)	2.36 (0.26-21.02)	0.95 (0.81-1.12)	0.857 (0.839-0.897)	0	0.00	6	1.70	0.00 (0.00-26.46)	98.30 (96.33-99.37)	NA	1.02 (1.00-1.03)	0.951 (0.951-0.965)	0	0.00	4	1.42	0.00 (0.00-60.24)	98.58 (96.41-99.61)	NA	1.01 (1.00-1.03)	0.972 (0.972-0.987)
	Diarrhea	1	7.69	6	6.52	7.69 (0.19-36.03)	93.48 (86.34-97.57)	1.18 (0.15-9.03)	0.99 (0.84-1.17)	0.829 (0.811-0.883)	0	0.00	20	5.68	0.00 (0.00-26.46)	94.32 (91.36-96.50)	NA	1.06 (1.03-1.09)	0.912 (0.912-0.930)	0	0.00	2	0.71	0.00 (0.00-60.24)	99.29 (97.46-99.91)	NA	1.01 (1.00-1.02)	0.979 (0.979-0.990)
	Shortness of breath	1	7.69	21	22.83	7.69 (0.19-36.03)	77.17 (67.25-85.28)	0.34 (0.05-2.30)	1.20 (0.99-1.45)	0.686 (0.668-0.754)	5	41.67	112	31.82	41.67 (15.17-72.33)	68.18 (63.04-73.02)	1.31 (0.66-2.60)	0.86 (0.53-1.39)	0.673 (0.657-0.692)	1	25.00	81	28.72	25.00 (0.63-80.59)	71.28 (65.61-76.49)	0.87 (0.16-4.80)	1.05 (0.59-1.86)	0.706 (0.700-0.721)
	Clinical signs of pneumonia (auscultatory)	0	0.00	18	19.57	0.00 (0.00-24.71)	80.43 (70.85-87.97)	NA	1.24 (1.12-1.38)	0.705 (0.705-0.768)	2	16.67	67	19.03	16.67 (2.09-48.41)	80.97 (76.47-84.93)	0.88 (0.24-3.16)	1.03 (0.80-1.33)	0.788 (0.779-0.809)	1	25.00	69	24.47	25.00 (0.63-80.59)	75.53 (70.08-80.43)	1.02 (0.18-5.65)	0.99 (0.56-1.76)	0.748 (0.742-0.763)
**Vaccination status**	Vaccinated against seasonal flu ever before	0	0.00	0	0.00	NA	NA	NA	NA	NA	1	8.33	22	6.25	8.33 (0.21-38.48)	93.75 (90.69-96.04)	1.33 (0.20-9.09)	0.98 (0.82-1.16)	0.909 (0.904-0.929)	2	50.00	53	18.79	50.00 (6.76-93.24)	81.21 (76.15-85.59)	2.66 (0.97-7.30)	0.62 (0.23-1.64)	0.808 (0.796-0.819)
	Vaccinated against the flu last year	0	0.00	0	0.00	NA	NA	NA	NA	NA	1	8.33	18	5.11	8.33 (0.21-38.48)	94.89 (92.04-96.94)	1.63 (0.24-11.22)	0.97 (0.81-1.15)	0.920 (0.915-0.940)	2	50.00	47	16.67	50.00 (6.76-93.24)	83.33 (78.46-87.49)	3.00 (1.09-8.27)	0.60 (0.22-1.60)	0.829 (0.817-0.840)

	Vaccinated against the flu this year	0	0.00	0	0.00	NA	NA	NA	NA	NA	1	8.33	18	5.11	8.33 (0.21-38.48)	94.89 (92.04-96.94)	1.63 (0.24-11.22)	0.97 (0.81-1.15)	0.920 (0.915-0.940)	2	50.00	50	17.73	50.00 (6.76-93.24)	82.27 (77.30-86.54)	2.82 (1.03-7.76)	0.61 (0.23-1.62)	0.818 (0.807-0.830)
	Vaccinated against COVID-19 in a timely manner	2	15.38	25	27.17	15.38 (1.92-45.45)	72.83 (62.55-81.58)	0.57 (0.15-2.12)	1.16 (0.89-1.51)	0.657 (0.626-0.728)	7	58.33	207	58.81	58.33 (27.67-84.83)	41.19 (36.00-46.53)	0.99 (0.61-1.61)	1.01 (0.51-2.00)	0.418 (0.398-0.434)	4	100.00	224	79.43	100.00 (39.76-100.00)	20.57 (16.00-25.76)	1.26 (1.19-1.34)	NA	0.217 (0.200-0.217)
**Chronic disease*** **	Hypertension	0	0.00	0	0.00	NA	NA	NA	NA	NA	4	33.33	79	22.44	33.33 (9.92-65.11)	77.56 (72.83-81.81)	1.49 (0.65-3.38)	0.86 (0.57-1.29)	0.761 (0.747-0.781)	3	75.00	257	91.13	75.00 (19.41-99.37)	8.87 (5.82-12.81)	0.82 (0.47-1.45)	2.82 (0.50-16.04)	0.098 (0.083-0.105)
	Myocardial infarction	0	0.00	0	0.00	NA	NA	NA	NA	NA	0	0.00	2	0.57	0.00 (0.00-26.46)	99.43 (97.96-99.93)	NA	1.01 (1.00-1.01)	0.962 (0.962-0.970)	1	25.00	19	6.74	25.00 (0.63-80.59)	93.26 (89.68-95.90)	3.71 (0.64-21.40)	0.80 (0.46-1.42)	0.923 (0.916-0.938)
	Cardiac insuficience	0	0.00	0	0.00	NA	NA	NA	NA	NA	0	0.00	3	0.85	0.00 (0.00-26.46)	99.15 (97.53-99.82)	NA	1.01 (1.00-1.01)	0.959 (0.959-0.970)	1	25.00	22	7.80	25.00 (0.63-80.59)	92.20 (88.43-95.05)	3.20 (0.56-18.33)	0.81 (0.46-1.43)	0.913 (0.906-0.927)
	Angina pectoris	0	0.00	0	0.00	NA	NA	NA	NA	NA	0	0.00	3	0.85	0.00 (0.00-26.46)	99.15 (97.53-99.82)	NA	1.01 (1.00-1.01)	0.959 (0.959-0.970)	1	25.00	12	4.26	25.00 (0.63-80.59)	95.74 (92.68-97.78)	5.88 (0.99-35.03)	0.78 (0.44-1.38)	0.948 (0.941-0.962)
	Arrhythmia	0	0.00	0	0.00	NA	NA	NA	NA	NA	1	8.33	11	3.13	8.33 (0.21-38.48)	96.88 (94.48-98.43)	2.67 (0.37-19.02)	0.95 (0.80-1.12)	0.940 (0.934-0.958)	0	0.00	20	7.09	0.00 (0.00-60.24)	92.91 (89.26-95.61)	NA	1.08 (1.04-1.11)	0.916 (0.916-0.933)
	Stroke	0	0.00	0	0.00	NA	NA	NA	NA	NA	0	0.00	1	0.28	0.00 (0.00-26.46)	99.72 (98.43-99.99)	NA	1.01 (1.00-1.01)	0.964 (0.964-0.969)	0	0.00	11	3.90	0.00 (0.00-60.24)	96.10 (93.13-98.04)	NA	1.04 (1.02-1.07)	0.948 (0.948-0.964)
	Asthma	0	0.00	8	8.70	0.00 (0.00-24.71)	91.30 (83.58-96.17)	NA	1.10 (1.03-1.17)	0.800 (0,800-0.856)	2	16.67	24	6.82	16.67 (2.09-48.41)	93.18 (90.02-95.58)	2.44 (0.65-9.18)	0.89 (0.69-1.15)	0.907 (0.898-0.927)	1	25.00	28	9.93	25.00 (0.63-80.59)	90.07 (85.97-93.30)	2.52 (0.44-14.25)	0.83 (0.47-1.47)	0.892 (0.885-0.906)
	Diabetes mellitus type 1	0	0.00	0	0.00	NA	NA	NA	NA	NA	1	8.33	5	1.42	8.33 (0.21-38.48)	98.58 (96.72-99.54)	5.87 (0.74-46.42)	0.93 (0.78-1.10)	0.956 (0.951-0.971)	0	0.00	5	1.77	0.00 (0.00-60.24)	98.23 (95.91-99.42)	NA	1.02 (1.00-1.03)	0.969 (0.969-0.984)
	Diabetes mellitus type 2	0	0.00	0	0.00	NA	NA	NA	NA	NA	0	0.00	12	3.41	0.00 (0.00-26.46)	96.59 (94.1	NA	1.04 (1.0	0.934 (0.934-0.951)	0	0.00	54	19.15	0.00 (0.00-60.24)	80.85 (75.7	NA	1.24 (1.17-	0.797 (0.797-0.814)

																2-98.23)		2-1.06)							7-85.27)		1.31)	
	Obesity	0	0.00	2	2.17	0.00 (0.00-24.71)	97.83 (92.37-99.74)	NA	1.02 (0.99-1.05)	0.857 (0.857-0.888)	2	16.67	23	6.53	16.67 (2.09-48.41)	93.47 (90.36-95.81)	2.55 (0.68-9.60)	0.89 (0.69-1.15)	0.909 (0.900-0.929)	1	25.00	22	7.80	25.00 (0.63-80.59)	92.20 (88.43-95.05)	3.20 (0.56-18.33)	0.81 (0.46-1.43)	0.913 (0.906-0.927)
	Other	1	7.69	6	6.52	7.69 (0.19-36.03)	93.48 (86.34-97.57)	1.18 (0.15-9.03)	0.99 (0.84-1.17)	0.829 (0.811-0.883)	4	33.33	78	22.16	33.33 (9.92-65.11)	77.84 (73.13-82.07)	1.50 (0.66-3.43)	0.86 (0.57-1.28)	0.764 (0.749-0.784)	1	25.00	116	41.13	25.00 (0.63-80.59)	58.87 (52.88-64.67)	0.61 (0.11-3.34)	1.27 (0.72-2.26)	0.584 (0.577-0.599)
	Without chronic diseases	12	92.31	78	84.78	92.31 (63.97-99.81)	15.22 (8.58-24.21)	1.09 (0.91-1.30)	0.51 (0.07-3.53)	0.248 (0.182-0.266)	4	33.33	209	59.38	33.33 (9.92-65.11)	40.62 (35.45-45.96)	0.56 (0.25-1.26)	1.64 (1.08-2.50)	0.404 (0.389-0.424)	1	25.00	14	4.96	25.00 (0.63-80.59)	95.04 (91.81-97.26)	5.04 (0.86-29.64)	0.79 (0.45-1.39)	0.941 (0.934-0.955)
**Children aged 7 to 14 in the patient's family**		1	7.69	3	3.26	7.69 (0.19-36.03)	96.74 (90.77-99.32)	2.36 (0.26-21.02)	0.95 (0.81-1.12)	0.857 (0.839-0.897)	1	8.33	45	12.78	8.33 (0.21-38.48)	87.22 (83.27-90.52)	0.65 (0.10-4.34)	1.05 (0.88-1.25)	0.846 (0.841-0.866)	0	0.00	5	1.77	0.00 (0.00-60.24)	98.23 (95.91-99.42)	NA	1.02 (1.00-1.03)	0.969 (0.969-0.984)
**Children aged 15 to 18 in the patient's family**		0	0.00	2	2.17	0.00 (0.00-24.71)	97.83 (92.37-99.74)	NA	1.02 (0.99-1.05)	0.857 (0.857-0.888)	1	8.33	41	11.65	8.33 (0.21-38.48)	88.35 (84.53-91.51)	0.72 (0.11-4.78)	1.04 (0.87-1.24)	0.857 (0.852-0.877)	0	0.00	5	1.77	0.00 (0.00-60.24)	98.23 (95.91-99.42)	NA	1.02 (1.00-1.03)	0.969 (0.969-0.984)
**Contact with someone who had flu-like symptoms seven days before testing**		9	69.23	83	90.22	69.23 (38.57-90.91)	9.78 (4.57-17.76)	0.77 (0.53-1.11)	3.15 (1.13-8.76)	0.171 (0.109-0.221)	12	100.00	273	77.56	100.00 (73.54-100.00)	22.44 (18.19-27.17)	1.29 (1.22-1.36)	NA	0.250 (0.231-0.250)	4	100.00	221	78.37	100.00 (39.76-100.00)	21.63 (16.97-26.90)	1.28 (1.20-1.36)	NA	0.227 (0.210-0.227)
**Smoking**		2	15.38	7	7.61	15.38 (1.92-45.45)	92.39 (84.95-96.89)	2.02 (0.47-8.71)	0.92 (0.72-1.16)	0.829 (0.797-0.887)	2	16.67	50	14.20	16.67 (2.09-48.41)	85.80 (81.71-89.27)	1.17 (0.32-4.27)	0.97 (0.75-1.26)	0.835 (0.826-0.856)	0	0.00	11	3.90	0.00 (0.00-60.24)	96.10 (93.13-98.04)	NA	1.04 (1.02-1.07)	0.948 (0.948-0.964)
**Alcohol consumption**		0	0.00	3	3.26	0.00 (0.00-24.71)	96.74 (90.77-99.32)	NA	1.03 (1.00-1.07)	0.848 (0.848-0.886)	0	0.00	12	3.41	0.00 (0.00-26.46)	96.59 (94.12-98.23)	NA	1.04 (1.02-1.06)	0.934 (0.934-0.951)	0	0.00	7	2.48	0.00 (0.00-60.24)	97.52 (94.95-99.00)	NA	1.03 (1.01-1.04)	0.962 (0.962-0.977)
**Use of buses for transportation purposes**		10	76.92	83	90.22	76.92 (46.19-94.96)	9.78 (4.57-17.76)	0.85 (0.63-1.16)	2.36 (0.73-7.60)	0.819 (0.778-0.882)	10	83.33	292	82.95	83.33 (51.59-97.91)	17.05 (13.27-21.39)	1.00 (0.78-1.30)	0.98 (0.27-3.54)	0.192 (0.171-0.201)	4	100.00	258	91.49	100.00 (39.76-100.00)	8.51 (5.53-12.40)	1.09 (1.05-1.13)	NA	0.098 (0.081-0.098)
**Use of TAXI for transportation purposes**		7	53.85	74	80.43	53.85 (25.13-80.78)	19.57 (12.03-29.15)	0.67 (0.40-1.12)	2.36 (1.15-4.84)	0.238 (0.174-0.299)	11	91.67	296	84.09	91.67 (61.52-99.79)	15.91 (12.25-20.16)	1.09 (0.91-1.30)	0.52 (0.08-3.47)	0.184 (0.164-0.189)	3	75.00	249	88.30	75.00 (19.41-99.37)	11.70 (8.19-16.04)	0.85 (0.48-1.50)	2.14 (0.38-12.02)	0.126 (0.111-0.132)

**Period of inclusion in the study**	1.12.23 - 14.2.2024.	12	92.31	49	53.26	92.31 (63.97 - 99.81)	46.74 (36.26-57.44)	1.73 (1.35-2.22)	0.16 (0.02-1.10)	0.524 (0.454-0.542)	9	75.00	244	69.32	75.00 (42.81-94.51)	30.68 (25.90-35.79)	1.08 (0.77-1.51)	0.81 (0.30-2.20)	0.321 (0.301-0.333)	4	100.00	148	52.48	100.00 (39.76-100.00)	47.52 (41.56-53.52)	1.91 (1.71-2.13)	NA	0.483 (0.466-0.483)
	15.2.2024 - 30.4.2024.	1	7.69	43	46.74	7.69 (0.19-36.03)	53.26 (42.56-63.74)	0.16 (0.02-1.10)	1.73 (1.35-2.22)	0.476 (0.458-0.546)	3	25.00	108	30.68	25.00 (5.49-57.19)	69.32 (64.21-74.10)	0.81 (0.30-2.20)	1.08 (0.77-1.51)	0.679 (0.667-0.699)	0	0.00	134	47.52	0.00 (0.00-60.24)	52.48 (46.48-58.44)	NA	1.91 (1.71-2.13)	0.517 (0.517-0.534)

*one patient could have one or more symptoms, simultaneously; **including feverishness; ***one patient could have one or more comorbidities, simultaneously; Se: sensitivitiy; Sp: specificity; LR+: positive likelihood ratio; LR-: negative likelihood ratio.
