# Supplementary material for: Diagnostic Significance of Influenza Symptoms and Signs, and Their Variation by Type/Subtype, in Outpatients Aged ≥ 15 Years: Novi Sad, Serbia
Source: Viruses. 2025 Feb 16;17(2):272. doi: 10.3390/v17020272 (PMC11860240; doi:10.3390/v17020272)
Supplement: Supplementary file 1 [file viruses-17-00272-s001.zip › Table S3.pdf]

Table S3. Differences between types of influenza regarding characteristics of participants in the 2022/23 season

Characteristics		A(H1N1) pdm09 (n=36)	%	A(H3N2) (n=11)	%	B (n=36)	%	A(H1N1) pdm09 vs. A (H3N2)	A(H1N1) pdm09 vs. B	A (H3N2) vs. B
**Age (years)**	15-29	9	25.00	4	36.36	15	41.67	0.4658	0.1363	0.7560
	30-64	22	61.11	7	63.64	19	52.78	0.8812	0.4785	0.5305
	≥ 65	5	13.89	0	0.00	2	5.56	0.1958	0.2362	0.4291
**Symptoms and signs**	Fever (≥ 38 °C)*	32	88.89	10	90.91	31	86.11	0.8514	0.7232	0.6796
	Cough	32	88.89	11	100.00	28	77.78	0.2531	0.2091	0.0895
	Sudden onset of symptoms	30	83.33	6	54.55	29	80.56	**0.0509**	0.7616	0.0867
	Headache	21	58.33	10	90.91	20	55.56	**0.0483**	0.8137	**0.0346**
	Dizziness	9	25.00	6	54.55	8	22.22	0.0687	0.7827	**0.0423**
	Sore throat	28	77.78	6	54.55	31	86.11	0.1359	0.3616	**0.0268**
	Nasal congestion	31	86.11	8	72.73	33	91.67	0.3066	0.4560	0.1031
	Myalgia	29	80.56	5	45.45	26	72.22	**0.0242**	0.4080	0.1047
	Malaise	28	77.78	8	72.73	34	94.44	0.7320	**0.0424**	**0.0432**
	Chills	21	58.33	5	45.45	28	77.78	0.4569	0.0788	**0.0423**
	Loss of appetite	8	22.22	2	18.18	10	27.78	0.7768	0.5885	0.5272
	Abdominal pain	4	11.11	0	0.00	4	11.11	0.2529	1.000	0.2529
	Nausea	3	8.33	0	0.00	2	5.56	0.3277	0.6462	0.4291
	Vomiting	5	13.89	0	0.00	2	5.56	0.1958	0.2362	0.4291
	Diarrhea	3	8.33	0	0.00	1	2.78	0.3277	0.3073	0.5803
	Shortness of breath	7	19.44	3	27.27	5	13.89	0.5827	0.5304	0.3066
	Clinical signs of pneumonia (auscultatory)	6	16.67	0	0.00	2	5.56	0.1515	0.1364	0.4291
**Vaccination status**	Vaccinated against seasonal flu ever before	2	5.56	1	9.09	3	8.33	0.6784	0.6462	0.9376
	Vaccinated against the flu last year	2	5.56	1	9.09	1	2.78	0.6784	0.5579	0.3694
	Vaccinated against the flu this year	2	5.56	2	18.18	1	2.78	0.1941	0.5579	0.0705
	Vaccinated against COVID-19 in a timely manner	14	38.89	6	54.55	15	41.67	0.3631	0.8113	0.4569
**Chronic disease**	Hypertension	10	27.78	3	27.27	6	16.67	0.9739	0.2602	0.4392
	Myocardial infarction	0	0.00	1	9.09	0	0.00	0.0705	NA	0.0705
	Cardiac insuficience	0	0.00	1	9.09	1	2.78	0.0705	0.3171	0.3694
	Angina pectoris	1	2.78	0	0.00	1	2.78	0.5803	1.000	0.5803
	Arrhythmia	1	2.78	0	0.00	1	2.78	0.5803	1.000	0.5803
	Stroke	2	5.56	0	0.00	0	0.00	0.4291	0.1542	NA
	Asthma	1	2.78	0	0.00	3	8.33	0.5803	0.3073	0.3277
	Diabetes mellitus type 1	0	0.00	0	0.00	1	2.78	NA	0.3171	0.5803
	Diabetes mellitus type 2	3	8.33	1	9.09	0	0.00	0.9376	0.0790	0.0705
	Obesity	3	8.33	0	0.00	1	2.78	0.3277	0.3073	0.5803
	Other	10	27.78	7	63.64	7	19.44	**0.0335**	0.4080	**0.0055**
	Without chronic diseases	21	58.33	4	36.36	22	61.11	0.2061	0.8113	0.1528

**Other variables**	Children aged 7-14 years in the family	10	27.78	3	27.27	12	33.33	0.9739	0.6117	0.7089
	Children aged 15-19 years in the family	5	13.89	1	9.09	2	5.56	0.6796	0.2362	0.6784
	Contact with someone who had flu-like symptoms seven days before testing	29	80.56	10	90.91	28	77.78	0.4290	0.7730	0.3379
	Smoking	12	33.33	4	36.36	4	11.11	0.8543	**0.0243**	0.0537
	Alcohol consumption	4	11.11	2	18.18	2	5.56	0.5429	0.3976	0.1941
	Use of buses for transportation purposes	26	72.22	6	54.55	28	77.78	0.2764	0.5885	0.1359
	Use of TAXI for transportation purposes	27	75.00	10	90.91	26	72.22	0.2643	0.7904	0.2049
	Confirmed between December 1, 2022 and February 14, 2023	28	77.78	7	63.64	14	38.89	0.3509	**0.0009**	0.1528
	Confirmed between February 15, 2023 and April 30, 2023	8	22.22	4	36.36	22	61.11	0.3517	**0.0009**	0.1528

*including feverishness; values that differ significantly ($p < 0.05$) are marked in bold; NA-not applicable.
