# Supplementary material for: Diagnostic Significance of Influenza Symptoms and Signs, and Their Variation by Type/Subtype, in Outpatients Aged ≥ 15 Years: Novi Sad, Serbia
Source: Viruses. 2025 Feb 16;17(2):272. doi: 10.3390/v17020272 (PMC11860240; doi:10.3390/v17020272)
Supplement: Supplementary file 1 [file viruses-17-00272-s001.zip › Table S4.pdf]

Table S4. Differences between types of influenza regarding characteristics of participants in the 2023/24 season

**Characteristics**		**A(H1N1) pdm09 (n=7)**	**%**	**A(H3N2) (n=19)**	**%**	**B (n=3)**	**%**	**A(H1N1) pdm09 vs. A (H3N2)**	**A(H1N1) pdm09 vs. B**	**A (H3N2) vs. B**
**Age (years)**	15-29	1	14.29	11	57.89	1	33.33	**0.0524**	0.5129	0.4379
	30-64	4	57.14	6	31.58	2	66.67	0.2439	0.7891	0.2513
	≥ 65	2	28.57	2	10.53	0	0.00	0.2675	0.3261	0.5647
**Symptoms and signs**	Fever (≥ 38 °C)*	6	85.71	18	94.74	3	100.00	0.4523	0.5126	0.7972
	Cough	7	100.00	19	100.00	3	100.00	NA	NA	NA
	Sudden onset of symptoms	3	42.86	15	78.95	2	66.67	0.0829	0.5127	0.6449
	Headache	5	71.43	14	73.68	3	100.00	0.9104	0.3261	0.3233
	Dizziness	2	28.57	0	0.00	0	0.00	**0.0174**	0.3261	NA
	Sore throat	4	57.14	15	78.95	1	33.,33	0.2755	0.5127	0.1072
	Nasal congestion	5	71.43	9	47.37	2	66.67	0.2844	0.8864	0.5438
	Myalgia	4	57.14	9	47.37	2	66.67	0.6648	0.7891	0.5438
	Malaise	5	71.43	15	78.95	3	100.00	0.6922	0.3261	0.3907
	Chills	3	42.86	3	15.79	0	0.00	0.1542	0.1985	0.4693
	Loss of appetite	2	28.57	2	10.53	0	0.00	0.2675	0.3261	0.5647
	Abdominal pain	0	0.00	2	10.53	0	0.00	0.3809	NA	0.5647
	Nausea	2	28.57	1	5.26	0	0.00	0.1056	0.3261	0.6912
	Vomiting	1	14.29	0	0.00	0	0.00	0.0994	0.5126	NA
	Diarrhea	1	14.29	0	0.00	0	0.00	0.0994	0.5126	NA
	Shortness of breath	2	28.57	3	15.79	2	66.67	0.4720	0.2850	0.0562
	Clinical signs of pneumonia (auscultatory)	0	0.00	2	10.53	1	33.33	0.3809	0.1267	0.2961
**Vaccination status**	Vaccinated against seasonal flu ever before	0	0.00	2	10.53	1	33.33	0.3809	0.1267	0.2961
	Vaccinated against the flu last year	0	0.00	2	10.53	1	33.33	0.3809	0.1267	0.2961
	Vaccinated against the flu this year	0	0.00	2	10.53	1	33.33	0.3809	0.1267	0.2961
	Vaccinated against COVID-19 in a timely manner	3	42.86	8	42.11	2	66.67	0.9731	0.5127	0.4379
**Chronic disease**	Hypertension	3	42.86	4	21.05	0	0.00	0.2755	0.1985	0.3907
	Myocardial infarction	1	14.29	0	0.00	0	0.00	0.0994	0.5126	NA
	Cardiac insuficience	1	14.29	0	0.00	0	0.00	0.0994	0.5126	NA
	Angina pectoris	1	14.29	0	0.00	0	0.00	0.0994	0.5126	NA
	Arrhythmia	0	0.00	1	5.26	0	0.00	0.5440	NA	0.6912

	Stroke	0	0.00	0	0.00	0	0.00	NA	NA	NA
	Asthma	0	0.00	3	15.79	0	0.00	0.2730	NA	0.4693
	Diabetes mellitus type 1	1	14.29	0	0.00	0	0.00	0.0994	0.5126	NA
	Diabetes mellitus type 2	0	0.00	0	0.00	0	0.00	NA	NA	NA
	Obesity	2	28.57	1	5.26	0	0.00	0.1056	0.3261	0.6912
	Other	3	42.86	3	15.79	0	0.00	0.1542	0.1985	0.4693
	Without chronic diseases	2	28.57	12	63.16	3	100.00	0.1238	**0.0495**	0.2135
**Other variables**	Children aged 7-14 years in the family	1	14.29	1	5.26	0	0.00	0.4523	0.5126	0.6912
	Children aged 15-19 years in the family	0	0.00	0	0.00	1	33.33	NA	0.1267	**0.0119**
	Contact with someone who had flu-like symptoms seven days before testing	6	85.71	16	84.21	3	100.00	0.9265	0.5126	0.4693
	Smoking	2	28.57	2	10.53	0	0.00	0.2675	0.3261	0.5647
	Alcohol consumption	0	0.00	0	0.00	0	0.00	NA	NA	NA
	Use of buses for transportation purposes	6	85.71	15	78.95	3	100.00	0.7036	0.5126	0.3907
	Use of TAXI for transportation purposes	6	85.71	13	68.42	2	66.67	0.3873	0.5129	0.9529
	Confirmed between December 1, 2023 and February 14, 2024	6	85.71	19	100.00	0	0.00	0.0994	**0.0162**	**< 0.0001**
	Confirmed between February 15, 2024 and April 30, 2024	1	14.29	0	0.00	3	100.00	0.0994	**0.0162**	**< 0.0001**

*including feverishness; values that differ significantly ($p < 0.05$) are marked in bold; NA-not applicable.
